# Modulation of Plant and Fungal Gene Expression Upon Cd Exposure and Symbiosis in Ericoid Mycorrhizal *Vaccinium myrtillus*

**DOI:** 10.3389/fmicb.2020.00341

**Published:** 2020-03-09

**Authors:** Salvatore Casarrubia, Elena Martino, Stefania Daghino, Annegret Kohler, Emmanuelle Morin, Hassine-Radhouane Khouja, Claude Murat, Kerrie W. Barry, Erika A. Lindquist, Francis M. Martin, Silvia Perotto

**Affiliations:** ^1^Department of Life Sciences and Systems Biology, University of Turin, Turin, Italy; ^2^Université de Lorraine, INRAE, UMR Interactions Arbres/Microorganismes, Centre INRAE Grand Est Nancy, Champenoux, France; ^3^U.S. Department of Energy Joint Genome Institute, Walnut Creek, CA, United States; ^4^Beijing Advanced Innovation Center for Tree Breeding by Molecular Design, Beijing Forestry University, Beijing, China

**Keywords:** Cd tolerance, transcriptomics, ericoid mycorrhiza, *Oidiodendron maius*, *Vaccinium myrtillus*

## Abstract

The success of Ericaceae in stressful habitats enriched in heavy metals has been ascribed to the distinctive abilities of their mycorrhizal fungal partners to withstand heavy metal stress and to enhance metal tolerance in the host plant. Whereas heavy metal tolerance has been extensively investigated in some ericoid mycorrhizal (ERM) fungi, the molecular and cellular mechanisms that extend tolerance to the host plant are currently unknown. Here, we show a reduced Cd content in Cd-exposed mycorrhizal roots of *Vaccinium myrtillus* colonized by a metal tolerant isolate of the fungus *Oidiodendron maius* as compared to non-mycorrhizal roots. To better understand this phenotype, we applied Next Generation Sequencing technologies to analyze gene expression in *V. myrtillus* and *O. maius* Zn grown under normal and Cd-stressed conditions, in the free living and in the mycorrhizal status. The results clearly showed that Cd had a stronger impact on plant gene expression than symbiosis, whereas fungal gene expression was mainly regulated by symbiosis. The higher abundance of transcripts coding for stress related proteins in non-mycorrhizal roots may be related to the higher Cd content. Regulated plant metal transporters have been identified that may play a role in reducing Cd content in mycorrhizal roots exposed to this metal.

## Introduction

Cadmium (Cd) is an extremely dangerous pollutant due to its high toxicity and persistence in the ecosystems. Cd can be easily taken up by plant roots thus entering the food chain and causing serious threats to animal and human health (Pinto et al., 2004; Peralta-Videa et al., 2009). Cd adversely affects plant growth and functioning even at low concentrations, with evident symptoms of chlorosis and imbalanced water and nutrients uptake (Küpper and Andresen, 2016). Due to its chemical similarity with trace elements – such as zinc (Zn), iron (Fe), and calcium (Ca) – and its high affinity for protein sulfhydryl groups, Cd can displace divalent cations and interfere with protein functioning. Although Cd does not participate directly to the generation of reactive oxygen species (ROS), Cd exposure leads to lipid and protein peroxidation and DNA damage likely because it interferes with the scavenging defense system involved in redox homeostasis (Bertin and Averbeck, 2006; Küpper and Andresen, 2016). Moreover, Cd is mutagenic because it interferes with the cellular mechanisms of DNA damage repair (Giaginis et al., 2006). In addition to oxidative stress and genotoxicity, Cd-induced effects in plants include inhibition of the photosynthetic apparatus and of root metabolism.

Extracellular avoidance mechanisms, such as secretion of chelating compounds or immobilization on cell wall components, may effectively reduce heavy metal accumulation in the plant cytoplasm (Sanità di Toppi and Gabbrielli, 1999). In addition, plants have a range of enzymatic and non-enzymatic scavenging mechanisms to avoid the deleterious effects of Cd within the cell, with the production of active metabolites such as glutathione, metallothioneins, and phytochelatins. These thiol-containing ligands can prevent uncontrolled Cd binding to non-target sites and are likely involved in metal sequestration into the vacuole (Clemens, 2006a; Küpper and Andresen, 2016). On the other hand, enzymes such as superoxide dismutase, catalase, ascorbate peroxidase, peroxidases, and antioxidant secondary metabolites (e.g., ascorbic acid, carotenoids, tocopherols) can buffer increased concentrations of intracellular ROS (Das and Roychoudhury, 2014).

In addition to these mechanisms, most plants can also cope with heavy metals by taking advantage of beneficial interactions with soil microorganisms. Among them, the symbiotic association with mycorrhizal fungi can increase the effectiveness of both avoidance and tolerance mechanisms against heavy metals (Luo et al., 2014; Ferrol et al., 2016; Ruytinx et al., 2016). Mycorrhizal fungi have been suggested to increase heavy metal tolerance of their host plant by different mechanisms, such as: (i) general health and growth improvement due to increased nutrient uptake, (ii) chelation/sequestration of toxic metals by external fungal structures (e.g., cell walls) or subcellular compartments (e.g., vacuoles and/or endoplasmic reticulum), (iii) regulation of plant metal import-export fluxes, (iv) induction of enzymatic and non-enzymatic mechanisms that allow plants to neutralize ROS produced by heavy metals (Garg and Bhandari, 2014; Luo et al., 2014; Ferrol et al., 2016).

Ericoid mycorrhiza (ERM) is an endosymbiosis that involves plants in the family Ericaceae and fungi mainly in the Leotiomycetes (Ascomycetes) and some Sebacinales (Basidiomycetes) (Perotto et al., 2012). Natural soils colonized by ericaceous plants are generally acidic, thus facilitating mobilization of heavy metals (Read, 1996; Cairney and Meharg, 2003). Bradley et al. (1981, 1982) first demonstrated that ericaceous plants can survive in metal-polluted soil conditions because of their association with ERM fungi. It was later demonstrated that ERM fungi from polluted soils can withstand high concentrations of Zn and Cd (Martino et al., 2000), and the ERM metal tolerant isolate *Oidiodendron maius* Zn was developed as a model system to investigate functional traits involved in fungal metal tolerance, with the release of the complete sequenced genome (Kohler et al., 2015). However, although some mechanisms of Cd tolerance have been identified in *O. maius* Zn (Daghino et al., 2016; Ruytinx et al., 2016), the molecular and cellular mechanisms that protect ERM plants and enable them to cope with toxic Cd levels are currently unknown. To start addressing this question, and more in general to understand how the plant and the fungus respond to Cd in symbiotic and asymbiotic conditions, we have grown *O. maius* Zn and its host plant *Vaccinium myrtillus* under control and Cd stressed conditions, and analyzed their metal content and transcriptomes in the free living and in the mycorrhizal status.

## Materials and Methods

### Fungal Strains

*Oidiodendron maius* Zn (hereafter *O. maius*) was isolated from the roots of *V. myrtillus* growing in experimental plots in the Niepolomice Forest (25 km northeast of Kraków, Poland), treated with industrial dusts containing high concentrations of heavy metals (Martino et al., 2000). This strain was deposited at the Mycotheca Universitatis Taurinensis collection (MUT1381; University of Turin, Italy) and at the American Type Culture Collection (ATCC MYA-4765; Manassas, VA, Untied States). The sequenced genome of *O. maius* is available on the Mycocosm website^1^.

### Plant and Fungal Growth in Control and Cd-Stressed Conditions

Non-mycorrhizal and mycorrhizal roots of *V. myrtillus* were obtained *in vitro* (Supplementary Figure S1) on a MMN medium (KH_2_PO_4_ 0.5 g L^–1^, BSA – bovine serum albumin, SIGMA – 0.1 g L^–1^, CaCl_2_^∗^2H_2_O 0.066 g L^–1^, NaCl 0.025 g L^–1^, MgSO_4_^∗^7H_2_O 0.15 g L^–1^, thiamine-HCl 0.1 g L^–1^, FeCl_3_^∗^6H_2_O 0.001 g L^–1^, agar 10 g L^–1^, final pH 4.7) as described in Kohler et al. (2015). In addition, for the Cd stressed condition, both *O. maius* and *V. myrtillus* were grown on the same medium containing 1 μM Cd (added as 3CdSO_4_^∗^8H_2_O). This Cd concentration was chosen because preliminary growth tests on non-mycorrhizal *V. myrtillus* seedlings had resulted, at higher Cd concentrations, in highly reduced plant growth and toxicity symptoms (Supplementary Figure S2). For RNA extraction, roots and aerial parts of mycorrhizal and non-mycorrhizal plants, as well as free living fungal mycelia, were sampled after 45 days of growth on control and Cd-amended medium (see next section). For ICP analysis, roots and aerial parts were processed as reported below.

### RNA-Seq and Data Analyses

For RNA extraction, root samples were weighted and immediately frozen in liquid nitrogen. Pooled samples were prepared containing roots of at least 10 *V. myrtillus* plants. Three pooled samples were analyzed for each treatment. RNA extraction and identification of mycorrhiza- and Cd-induced transcripts were performed as described in Kohler et al. (2015). Illumina HiSEq2500 sequencing (2 bp × 150 bp) was performed at the Joint Genome Institute (JGI, Walnut Creek, CA, United States). For the RNA-Seq experiment, three separate libraries were prepared from three biological replicates for each condition. CLC genomic workbench was used for trimming, alignment and identification of differentially expressed genes as described in Kohler et al. (2015). Filtered fastq files of plant-only samples were used as input for *de novo* assembly of *V. myrtillus* RNA contigs. Reads were assembled into consensus sequences using Trinity (ver. 2.1.1) (Grabherr et al., 2011). Contigs were annotated following BlastX searches against the *Arabidopsis thaliana*^2^ and the *Vaccinium macrocarpon* (Polashock et al., 2014) (http://bmcplantbiol.biomedcentral.com/articles/10.1186/1471-2229-14-165) databases.

The complete data sets have been deposited in NCBI’s Gene Expression Omnibus and are accessible through GEO Series accession numbers GSE119266 (*O. maius* data set) and GSE119554 (*V. myrtillus* data set).

### Measure of Cd Content

*Vaccinium myrtillus* roots and above-ground portions of mycorrhizal and non-mycorrhizal plants were collected and oven dried at 70°C until reaching a constant weight. The material was totally digested by a treatment in nitric acid 6M at 90°C for 1 h. After filtration with GF/C filters (Whatman) and appropriate dilution in milliQ water, the metal content was finally determined using Induced Coupled Plasma-Optical Emission Spectrometry (ICP-OES Vista MPX, Varian, Department of Earth Sciences, University of Turin, Italy). Controls made up of ultrapure water and nitric acid were also analyzed. The results are the mean of four biological replicates, obtained by pooling the material from 8 *V. myrtillus* plants.

### Bioinformatic Analyses

KEGG enrichment analyses was performed using the R package (“clusterProfiler”) in order to identify pathways that may be over-represented in the *V. myrtillus* and in the *O. maius* transcripts regulated by Cd and/or by the mycorrhizal symbiosis.

Putative transporters were predicted by Blastp against the Transporter Classification Database (TCDB)^3^ (Saier et al., 2016) with e-value ≤ 10^–5^, TCDB subfamily pfam domains were added to help annotate the potential transporters. Venn diagrams were built by using the web-tool http://bioinfogp.cnb.csic.es/tools/venny/index.html (Oliveros J. C., 2015).

Hierarchical clustering was performed by using hclust (R package Stats) with the Ward.D2 method, and the Pheatmap R package was used to build the heatmap plots and to perform the correlation analyses.

### Statistical Analyses

The significance of differences among the different treatments was statistically evaluated by ANOVA with Tukey’s pairwise comparison as *post hoc* test for multiple comparisons for data with normal distribution. Kruskal–Wallis with Bonferroni-corrected pairwise Mann–Whitney *post hoc* adjustment was used as statistical test for data with non-normal distribution. The differences were considered significant at a probability level of *p* < 0.05.

## Results

### Growth and Cd Accumulation

Symbiotic and asymbiotic growth of *V. myrtillus* seedlings and *O. maius* mycelium was measured on MMN medium amended with 1 μM Cd or unamended (control treatment). Preliminary growth tests with non-mycorrhizal *V. myrtillus* seedlings at higher Cd concentrations had resulted in severely impaired plant growth and toxicity symptoms (Supplementary Figure S2), so we chose this relatively low Cd concentration to investigate plant gene expression under comparable growth conditions in mycorrhizal and non-mycorrhizal roots.

**FIGURE 1:**
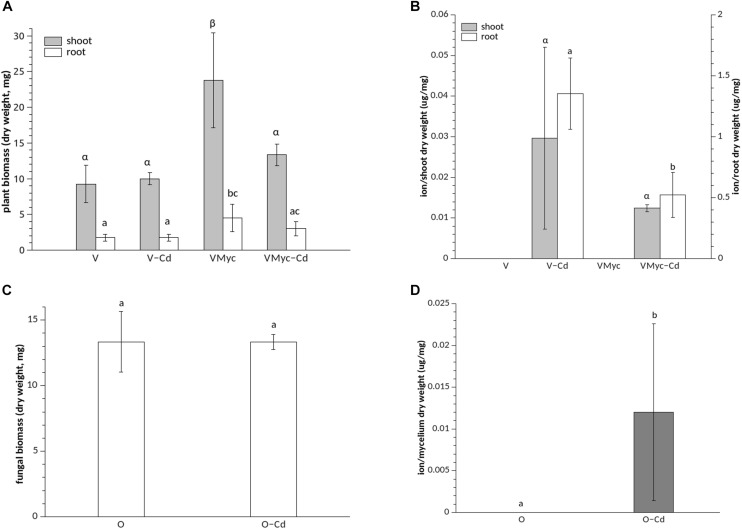
Growth and Cd concentrations in *Vaccinium myrtillus* roots and shoots and in *Oidiodendron maius* Zn. **(A)** Dry weight biomass of control (VMyc) and Cd-stressed mycorrhizal (VMyc-Cd) roots and shoots was compared with non-inoculated control (V) and Cd-stressed (V-Cd) roots and shoots of *V. myrtillus*. **(B)** Cd concentrations were quantified in the roots and in the shoots of mycorrhizal and non-mycorrhizal *V. myrtillus* seedlings, grown under control and Cd-stressed conditions. **(C)** Dry weight biomass of control and Cd-exposed *O. maius* Zn mycelium. **(D)** Cd concentrations in control and Cd-exposed *O. maius* Zn mycelium. Bars represent the mean ± SD, *n* = 4. Different letters (Greek letters for shoots and Latin letters for roots in A and B) indicate statistically significant differences (*p* < 0.05) according to ANOVA with Tukey’s as *post hoc* test.

In fact, under asymbiotic conditions, 1 μM Cd did not impair plant root biomass production, when compared with plants grown on control medium (Figure 1). Root biomass significantly increased in mycorrhizal *V. myrtillus*, irrespective of Cd exposure (Figure 1A). Similarly, growth of the aerial portions (stems and leaves) was not affected by Cd in asymbiotic *V. myrtillus* seedlings. A significant biomass increase of aerial parts, as compared to non-mycorrhizal plants, was only observed in mycorrhizal plants grown on control medium (Figure 1A). No differences in fungal biomass were observed when the free-living mycelium was grown on control or Cd amended medium (Figure 1C).

Mycorrhizal roots of *V. myrtillus* showed a significantly lower Cd content than non-mycorrhizal roots (Figure 1B). We also measured Fe content because Cd is known to interfere with Fe homeostasis by reducing Fe uptake (Clemens, 2006a). The Fe content was higher in mycorrhizal than in non-mycorrhizal *V. myrtillus* roots but, at the concentration used in this study, Cd did not interfere with Fe content in the plant roots or in the fungus (Supplementary Figure S3).

### Analysis of the *V. myrtillus* and *O. maius* Zn Transcriptomes

The same growth conditions described above were used to generate plant and fungal samples for RNAseq, as described in Kohler et al. (2015). Since the *V. myrtillus* genome sequence is not yet available, a *de novo* assembly of the *V. myrtillus* reads was generated and the 414.986 contigs were annotated by BlastX searches against the *Vaccinium macrocarpon* (Polashock et al., 2014) and the *Arabidopsis thaliana* proteomes.

Principal component analysis (PCA) was used to investigate plant and fungal transcriptomic distribution patterns in control and Cd-exposed conditions, in the free living and in the mycorrhizal status (Supplementary Figure S4). A clear Cd effect on the transcriptomic profile of mycorrhizal and non-mycorrhizal *V. myrtillus* roots could be observed (Supplementary Figure S4), whereas plant transcripts from mycorrhizal and non-mycorrhizal *V. myrtillus* roots partially overlapped in the control and the Cd-treated conditions (Supplementary Figure S4A). Concerning *O. maius*, the PCA indicated a stronger impact of the symbiotic status on the fungal transcriptomic profile than the Cd treatment, with a large overlapping between samples exposed to Cd or grown on control medium (Supplementary Figure S4B).

Three different pairwise comparisons were made to better dissect the responses of *V. myrtillus* and *O. maius* to Cd or to symbiosis. We investigated in particular: (1) the influence of Cd on plant and fungal gene expression in free-living conditions, (2) the influence of Cd on plant and fungal gene expression in symbiosis and (3) the influence of symbiosis on plant and fungal gene expression on control medium (see codes in Supplementary Table S1). For *V. myrtillus*, Supplementary Table S2 reports the total number of contigs identified with significant *p*-value (*p* < 0.05) and the percentage of protein hits found in the *A. thaliana* or in the *V. macrocarpon* databases. Since a higher percentage of translated *V. myrtillus* transcripts aligned with *A. thaliana* proteins for two out of the three comparisons (Supplementary Table S2), further analyses only considered *V. myrtillus* transcripts that found a protein hit on the *A. thaliana* database.

We considered FC > 2 as threshold values for the up-regulated transcripts and FC < 0.5 for the down-regulated transcripts (Table 1). For the three different pairwise comparisons, a complete list of the significantly regulated plant and fungal transcripts is reported in Supplementary Table S3.

**TABLE 1:** Number of transcripts regulated in *O. maius* Zn and *V. myrtillus.*

Organism	Comparison	Total n° of transcripts *p* < 0.05	Up-regulated genes FC > 2	Down-regulated genes FC < 0.5
*V. myrtillus* (V) *De novo*	V-Cd vs. V	227	109	37
	VMyc vs. V	217	181	2
	VMyc-Cd vs. VMyc	453	147	158
*O. maius* Zn (O)	O-Cd vs. O	501	120	14
	OMyc vs. O	7597 (7527 + 70MS^∗^)	1636 (1566 + 70MS)	1683
	OMyc-Cd vs. OMyc	743	176	198

*The total number of transcripts were counted using *p* < 0.05 as Baggerley’s test significance threshold. FC values higher than 2 and lower than 0.5 were considered to count up- and down-regulated transcripts in the different comparisons for both *O. maius* Zn and *V. myrtillus.* Only the transcripts annotated in the *A. thaliana* genome were considered (see Supplementary Table S3). ^∗^MS mycorrhizal specific.*

### Functional Annotation of the *V. myrtillus* and *O. maius* Transcriptomes

The Kyoto Encyclopedia of Genes and Genomes (KEGG) is widely used to identify the activation/inactivation of specific biochemical pathways. Functional KEGG enrichment analyses identified some over-represented pathways regulated by Cd and/or by symbiosis (Supplementary Figure S5). In non-mycorrhizal *V. myrtillus* roots, pathways up-regulated by Cd (V-Cd vs. V) were “Starch and sucrose metabolism,” “Protein processing in endoplasmic reticulum,” and “Cyanoamino acid metabolism.” In mycorrhizal roots, “Glutathione metabolism” and “Sesquiterpenoid and triterpenoid biosynthesis” pathways were specifically induced by Cd (VMyc-Cd vs. VMyc), together with enzymes involved in photosynthesis (Supplementary Figure S5).

In *O. maius* (Supplementary Figure S5), no over-represented metabolic pathways could be identified among the genes up-regulated by Cd in symbiosis (OMyc-Cd vs. OMyc), whereas KEGG pathways for “Cyclohexanone monooxygenase” and “Beta-lactamase” were up-regulated by Cd in the free-living mycelium (O-Cd vs. O). Among the fungal genes down-regulated by Cd in both symbiotic and asymbiotic conditions, Carbohydrate-Active Enzymes (CAZymes) were over-represented (Supplementary Figure S5).

### Shared and Specific *V. myrtillus* and *O. maius* Transcripts Induced by Cd Stress and Mycorrhizal Symbiosis

**FIGURE 2:**
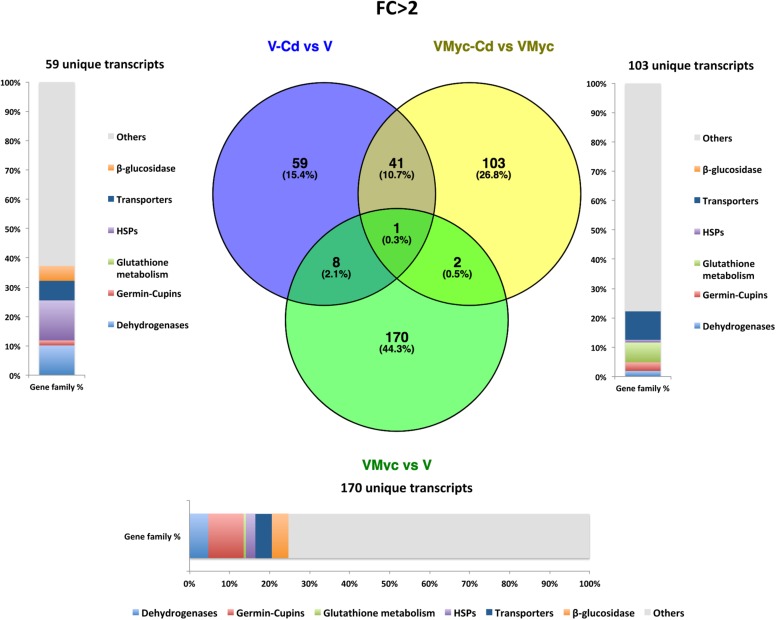
Shared and specific Cd- and symbiosis up-regulated *V. myrtillus* transcripts. Venn diagrams showing shared and specific up-regulated (FC > 2) transcripts of *V. myrtillus* in the following comparisons: mycorrhizal roots versus non-mycorrhizal roots on control medium (VMyc vs. V), mycorrhizal roots exposed to Cd versus mycorrhizal roots on control medium (VMyc-Cd vs. VMyc), non-mycorrhizal roots exposed to Cd versus non-mycorrhizal roots on control medium (V-Cd vs. V). Distribution of specifically up-regulated transcripts in six gene families: dehydrogenases, β-glucosidases, germin/germin-like proteins and cupins (germin-cupins), heat shock proteins (HSPs), transporters and proteins involved in glutathione metabolism is shown for up-regulated genes in the three pairwise comparisons. The chart represents abundance of transcripts in each family as percentage of the total number of specifically up-regulated transcripts.

**FIGURE 3:**
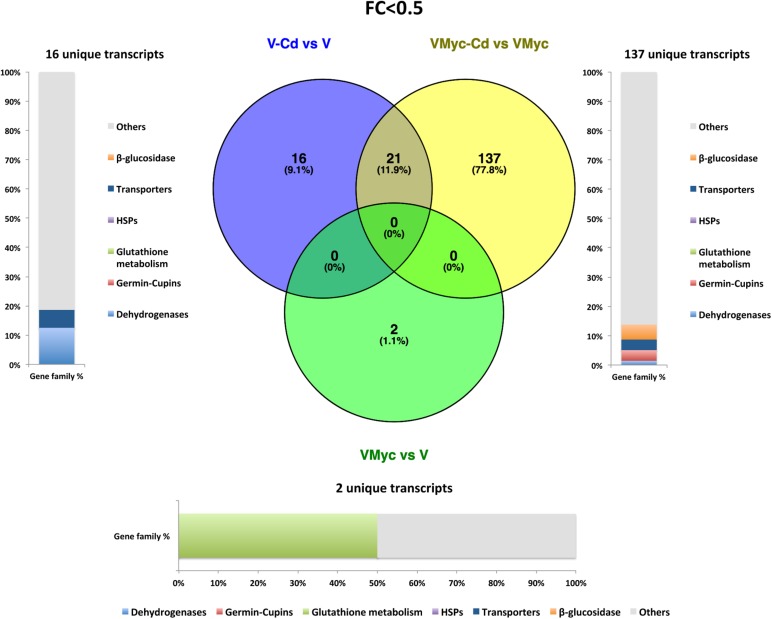
Shared and specific Cd- and symbiosis down-regulated *V. myrtillus* transcripts. Venn diagrams showing shared and specific down-regulated (FC < 0.5) transcripts of *V. myrtillus* in the following comparisons: mycorrhizal roots versus non-mycorrhizal roots on control medium (VMyc vs. V), mycorrhizal roots exposed to Cd versus mycorrhizal roots on control medium (VMyc-Cd vs. VMyc), non-mycorrhizal roots exposed to Cd versus non-mycorrhizal roots on control medium (V-Cd vs. V). Distribution of specifically down-regulated transcripts in six gene families: dehydrogenases, β-glucosidases, germin/germin-like proteins and cupins (germin-cupins), HSPs, transporters and proteins involved in glutathione metabolism is shown for down-regulated genes in the three pairwise comparisons. The chart represents abundance of transcripts in each family as percentage of the total number of specifically down-regulated transcripts.

**FIGURE 4:**
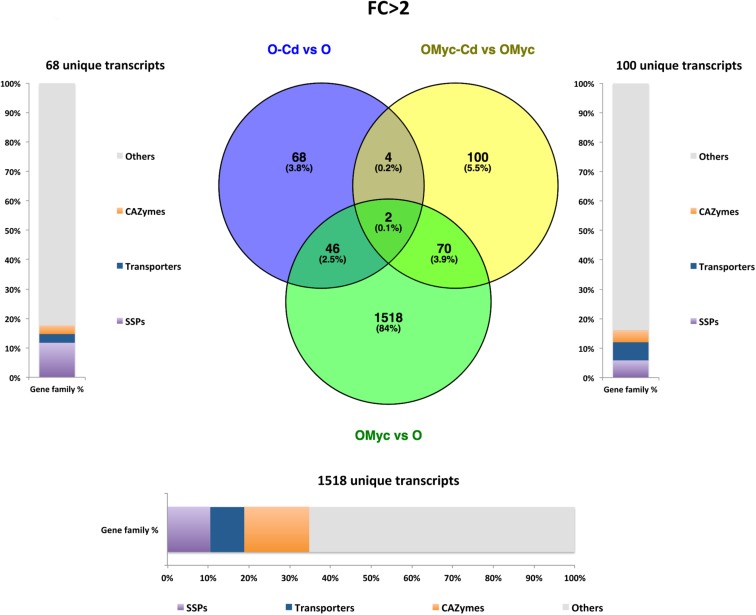
Shared and specific Cd- and symbiosis up-regulated *O. maius* Zn genes. Venn diagrams showing shared and specific up-regulated (FC > 2) transcripts of *O. maius Zn* in the following comparisons: mycorrhizal mycelium versus free living mycelium on control medium (OMyc vs. O), Cd-exposed free living mycelium versus free living mycelium on control medium (O-Cd vs. O), Cd-exposed mycorrhizal mycelium versus mycorrhizal mycelium on control medium (OMyc-Cd vs. OMyc). The distribution of specifically up-regulated genes in three gene families (transporters – TPs – CAZymes and Small Secreted Proteins – SSPs) is reported in the charts representing the abundance of transcripts in each family as percentage of the total number of specifically up-regulated transcripts.

**FIGURE 5:**
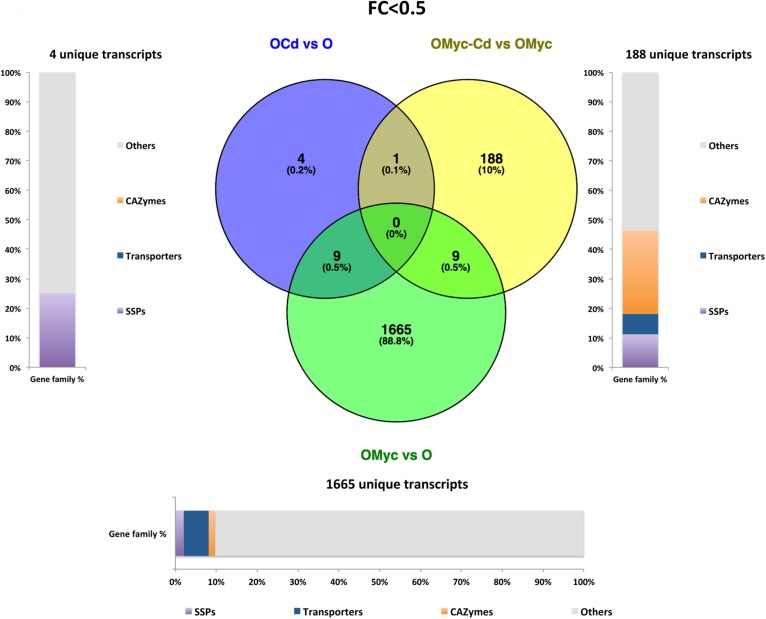
Shared and specific Cd- and symbiosis down-regulated *O. maius* Zn genes. Venn diagrams showing shared and specific down-regulated (FC < 0.5) transcripts of *O. maius Zn* in the following comparisons: mycorrhizal mycelium versus free living mycelium on control medium (OMyc vs. O), Cd-exposed free living mycelium versus free living mycelium on control medium (O-Cd vs. O), Cd-exposed mycorrhizal mycelium versus mycorrhizal mycelium on control medium (OMyc-Cd vs. OMyc). The distribution of specifically down-regulated genes in three gene families (transporters – TPs – CAZymes and Small Secreted Proteins – SSPs) is reported in the charts representing the abundance of transcripts in each family as percentage of the total number of specifically down-regulated transcripts.

Venn diagrams were built to visualize unique and shared up-regulated (FC > 2) and down-regulated (FC < 0.5) transcripts in both *V. myrtillus* (Figures 2, 3) and *O. maius* (Figures 4, 5). In *V. myrtillus*, symbiosis on control medium (VMyc vs. V) induced the highest number (170) of specific transcripts (Figure 2). In mycorrhizal roots, Cd exposure (VMyc-Cd vs. VMyc) led to a similar number of up-regulated (103) and down-regulated (137) specific transcripts (Figures 2, 3). Interestingly, most *V. myrtillus* transcripts involved in glutathione (GSH) metabolism were specifically induced by Cd in mycorrhizal roots (VMyc-Cd vs. VMyc, Figure 2), whereas heat shock proteins (HSPs) were particularly abundant among transcripts specifically up-regulated by Cd in non-mycorrhizal roots (V-Cd vs. V) (Figure 2). In mycorrhizal roots exposed to Cd, transcripts coding for ß-glucosidases (mainly belonging to Class 12) were specifically down-regulated, whereas these enzymes were up-regulated in the other two pairwise comparisons (Figures 2, 3). *V. myrtillus* transcripts coding for laccase, Cu/Zn superoxide dismutase, cupredoxin, expansin, glyoxal oxidase were also found to be specifically down-regulated in mycorrhizal roots exposed to Cd (Supplementary Table S3).

A Venn diagram built from the *O. maius* dataset illustrates the extensive changes of fungal gene expression specifically induced by symbiosis (OMyc vs. O), with 1518 and 1665 specific fungal genes being respectively up-regulated and down-regulated in mycorrhizal roots (Figures 4, 5). In the free-living mycelium, Cd exposure specifically up-regulated 68 and down-regulated four fungal genes (Figures 4, 5). Almost 12% of fungal transcripts specifically up-regulated by Cd coded for Small Secreted Proteins (SSPs) (Figure 4). When in symbiosis (OMyc-Cd vs. OMyc), Cd exposure specifically up-regulated 100 and down-regulated 188 fungal genes (Figures 4, 5). A large proportion of fungal transcripts specifically down-regulated by Cd in symbiosis coded for CAZymes (Figure 5).

### Influence of Cd and Symbiosis on *V. myrtillus* and *O. maius* Putative Transporter Families

**FIGURE 6:**
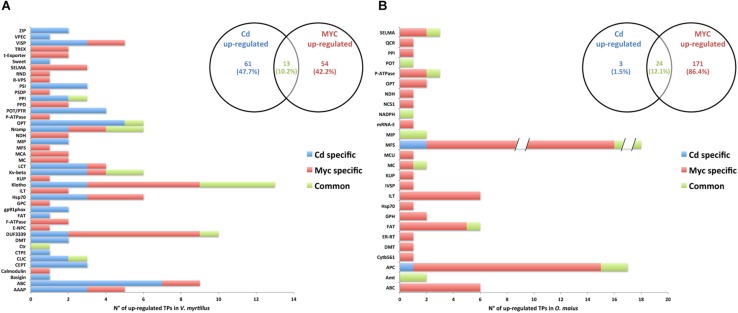
Putative transporter families up-regulated by Cd-exposure and/or by symbiosis in *V. myrtillus* and *O. maius* Zn. Plant and fungal putative TPs up-regulated by Cd-exposure and/or by the symbiosis were counted and their distribution among transcript/gene families is reported respectively for *V. myrtillus* **(A)** and *O. maius* Zn **(B)**. For a complete list of putative TPs, see Supplementary Tables S4, S5.

**FIGURE 7:**
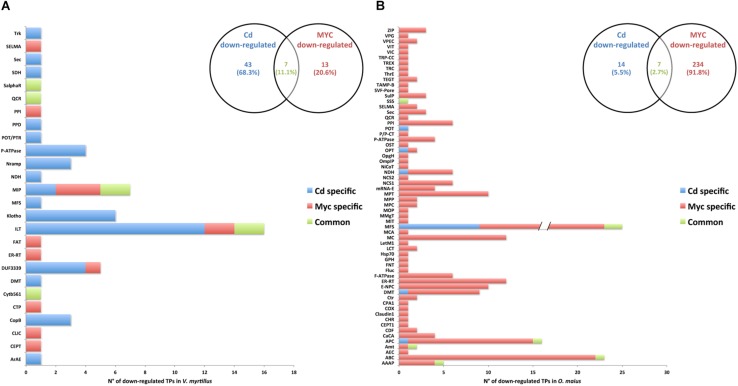
Putative transporter families down-regulated by Cd-exposure and/or by symbiosis in *V. myrtillus* and *O. maius* Zn. Plant and fungal putative TPs down-regulated by Cd-exposure and/or by the symbiosis were counted and their distribution among transcript/gene families is reported respectively for *V. myrtillus* **(A)** and *O. maius* Zn **(B)**. For a complete list of putative TPs, see Supplementary Tables S4, S5.

The ICP measurements showed a significantly lower Cd content in mycorrhizal roots of *V. myrtillus* than in non-mycorrhizal roots (Figure 1), a result that could be explained by either a decreased Cd influx or an increased Cd efflux. Given the crucial role of transporters (TPs) in ion movements across membranes, we investigated the protein family distribution and the expression patterns of significantly regulated *V. myrtillus* and *O. maiu*s TPs. Putative transporters were predicted by Blastp against the Transporter Classification Database, and the complete list of putative regulated TPs genes is provided for *V. myrtillus* (Supplementary Table S4) and *O. maiu*s (Supplementary Table S5). The distribution of these putative TPs families in the different conditions is summarized in the histograms in Figures 6, 7.

In *V. myrtillus*, many putative TPs were regulated by Cd rather than by symbiosis (Figures 6A, 7A). In particular, the percentage of up-regulated putative TPs was higher in mycorrhizal (9.71%) than in non-mycorrhizal (6.78%) roots exposed to Cd, or in mycorrhizal roots on control medium (4.12%). Among the 182 putative TPs regulated in *V. myrtillus*, 61 were specifically up-regulated and 43 were specifically down-regulated by Cd (Figures 6A, 7A and Supplementary Table S4). Cadmium-regulated TPs belong to 29 different families (Supplementary Table S4), the majority of up-regulated transcripts being in the ATP-binding Cassette (ABC) and in the Oligopeptide Transporter (OPT) families, and the majority of down-regulated transcripts being in the Iron/Lead Transporter (ILT) and the Klotho Auxiliary Protein (Klotho) families (Figures 6A, 7A and Supplementary Table S4). The mycorrhizal symbiosis specifically up-regulated 54 and down-regulated 13 *V. myrtillus* genes putatively involved in transport, the two most represented families being the Domain of Unknown Function 3339 (DUF3339) and the Klotho (Figures 6A, 7A).

The histograms in Figures 6B, 7B, clearly show that most (405/441) *O. maius* putative TPs genes were regulated by symbiosis, whereas Cd specifically regulated only 17 TP genes (3 up-regulated and 14 down-regulated genes) (Figures 6B, 7B and Supplementary Table S5).

**FIGURE 8:**
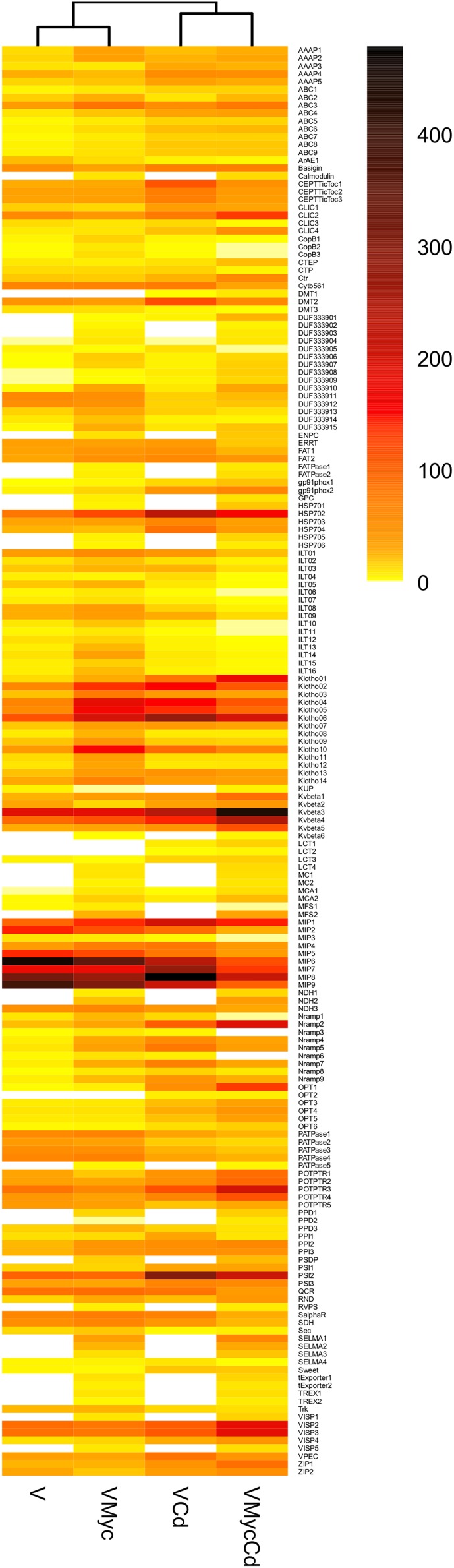
Cd-exposure and mycorrhizal effects on the expression pattern of *V. myrtillus* putative TPs families. The figure represents a heatmap visualization and single hierarchical clustering of the regulated (*p* < 0.05) putative TPs of *V. myrtillus*. Rows are ordered by TPs family composition whereas columns are ordered by hierarchical clustering. The intensity of the color is representative of the mean expression value of each transcript. On the right, the color-key expression code.

**FIGURE 9:**
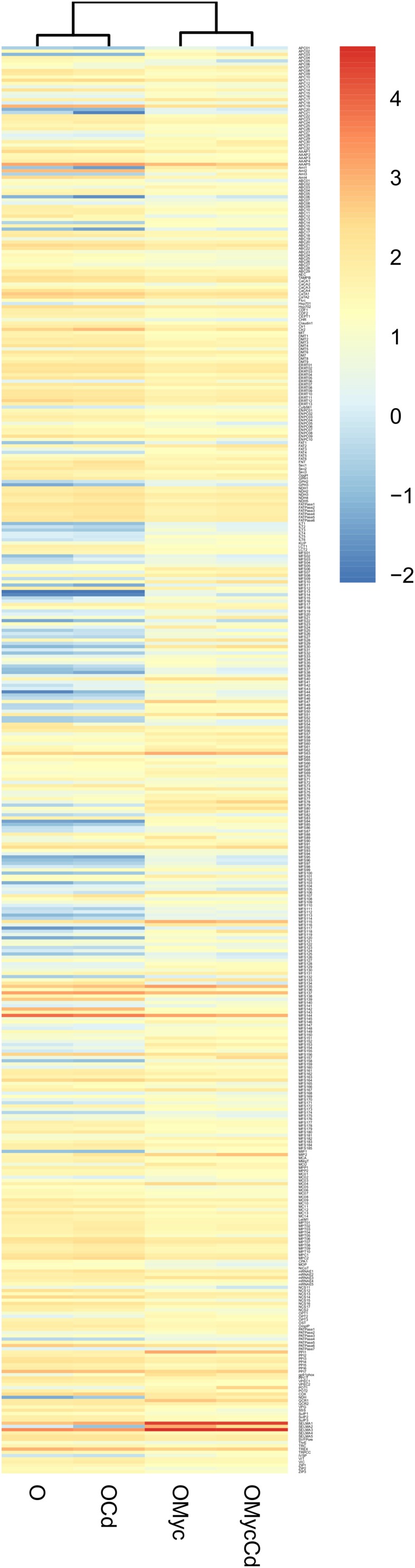
Cd-exposure and mycorrhizal effects on the expression pattern of *O. maius* Zn putative TPs families. The figure represents a heatmap visualization and single hierarchical clustering of the regulated (*p* < 0.05) putative TPs of *O. maius* Zn. Rows are ordered by TPs family composition whereas columns are ordered by hierarchical clustering. The intensity of the color is representative of the Log_10_ of mean expression value of each gene. On the right, the color-key expression code.

To further investigate the expression pattern of regulated plant and fungal putative TPs, hierarchical clustering of their expression values (as mean reads numbers) in the different conditions was performed for both *V. myrtillus* (Figure 8, and Supplementary Figure S6) and *O. maius* (Figure 9, and Supplementary Figure S7). In addition to a correlation analysis of TPs expression profiles, the resulting heatmaps allowed visual identification of TPs differentially expressed upon Cd exposure or in symbiosis. In particular, single hierarchical clustering (Figures 8, 9) allowed us to compare the expression profiles of members of a given gene family under different conditions, whereas double hierarchical clustering (Supplementary Figures S6, S7) identified groups of genes with similar expression profiles in a given condition. The heatmaps obtained for the *V. myrtillus* and *O. maius* putative TPs further demonstrate that the two experimental conditions (Cd exposure and symbiosis) had a very different impact on the regulation of TPs in the plant and in the fungus. According to correlation analyses of their TPs expression profiles, Cd-treated *V. myrtillus* plants were placed in the same cluster, irrespective of their mycorrhizal status (V-Cd and VMyc-Cd), whereas non-mycorrhizal and mycorrhizal plants (V and VMyc) grown on control medium grouped together in a separated cluster. This result indicates that transcriptomic changes induced by Cd in the plant (the “Cd-effect”) were stronger than those induced by symbiosis (the “Myc-effect”). Many transcripts up-regulated by Cd in mycorrhizal and non-mycorrhizal plants could be identified in the ABC superfamily and in the Metal Ion (Mn^2+^-iron) transporter and OPT families (Figure 8). Among the TPs induced by Cd in *V. myrtillus*, we also found two putative ZRT/IRT transporters (ZIP1 and ZIP2) (Figure 8). A copper transporter similar to *Arabidopsis* COPT3 was significantly up-regulated in mycorrhizal *V. myrtillus* roots exposed to Cd (Supplementary Table S4). Interestingly, some genes coding for Natural resistance-associated macrophage proteins (Nramp), members of the metal ion (Mn^2+^-iron) transporter family, were strongly down-regulated specifically in mycorrhizal roots exposed to Cd, as well as two members of the Copper Resistance Putative Porin (CopB) Family (Figure 8, see also Supplementary Table S4).

In contrast to the situation observed for *V. myrtillus*, correlation analyses of fungal TPs expression profiles in *O. maius* placed the two mycorrhizal conditions (OMyc-Cd and OMyc) in the same cluster, separate from the two free-living conditions (O-Cd and O). This result further indicates that symbiosis (the “Myc-effect”) had a stronger influence on the *O. maius* TPs expression profile than cadmium (the “Cd-effect”) (Figure 9, and Supplementary Figure S7). Several putative fungal TPs were regulated by symbiosis irrespective of Cd exposure (Figure 9, and Supplementary Table S5). Most of them belong to the Major facilitator superfamily (MFS) and where annotated as sugar transporters, but several amino acid permeases, two ammonium transporters and two Major Intrinsic Proteins (MIPs) were also regulated in the symbiotic fungus (Supplementary Table S5). Although none of the putative fungal TPs was specifically regulated by Cd in symbiosis, some genes up-regulated in mycorrhizal roots were more strongly induced when mycorrhizal plants were exposed to Cd (Supplementary Table S5). Among them were two ammonium transporters, some sugar transporters of the MFS superfamily, a heavy metal translocating P-type ATPase, a member of the Fatty Acid Transporter (FAT) Family and an amino acid permease (Figure 9, and Supplementary Table S5).

## Discussion

Mycorrhizal fungi can protect their host plant from metal toxicity and reports of increased metal tolerance of arbuscular and ectomycorrhizal plants, as compared to non-mycorrhizal plants, are numerous (e.g., see Colpaert et al., 2011; Ferrol et al., 2016). Similarly, increased metal tolerance was attributed to the fungal partner in ERM plants (Bradley et al., 1981, 1982). The cellular and molecular mechanisms that allow ERM fungi to withstand high heavy metal concentrations have been investigated and summarized in Daghino et al. (2016, 2019) and Ruytinx et al. (2016). These studies demonstrate that antioxidant enzymes, metal transporters, DNA damage repair proteins, molecular chaperons and polyamines biosynthesis are involved in the response of the metal tolerant ERM isolate *O. maius* Zn to metal toxicity. By contrast, the molecular mechanisms underlying the protective role of ERM fungi toward their host plant have not been investigated yet. To start addressing this aspect, we investigated gene expression profiles in mycorrhizal and non-mycorrhizal *V. myrtillus* and *O. maius*, in the absence and presence of Cd ions.

### Responses of *V. myrtillus* Roots to Cd Exposure

To investigate the influence of Cd on plant and fungal gene expression in symbiotic and asymbiotic conditions, we chose a relatively low (1 μM) Cd concentration to avoid excessive toxicity symptoms in asymbiotic *V. myrtillus* plants. At this Cd concentration, mycorrhizal roots of *V. myrtillus* displayed a significantly lower Cd content with respect to non-mycorrhizal roots, supporting the hypothesis that the ERM fungus may protect the host plant by reducing metal accumulation in the plant tissues. The influence of the mycorrhizal symbiosis on heavy metal accumulation in different plant species and organs has been extensively investigated, and contrasting data have been reported for both AM and ECM symbioses, with organs of mycorrhizal plant being found to accumulate either higher or lower amounts of heavy metals than non-mycorrhizal plants (Ouziad et al., 2005; Luo et al., 2014; Coninx et al., 2017; Shi et al., 2019).

Cadmium clearly affected the transcriptomic profile of both mycorrhizal and non-mycorrhizal *V. myrtillus* roots. The KEGG enrichment analysis showed, for non-mycorrhizal Cd-treated *V. myrtillus*, significant up-regulation of transcripts involved in the phenylpropanoid biosynthesis pathway. This pathway leads to the production of several aromatic secondary metabolites, including lignin precursors, flavonoids, coumarins, phenolic volatiles, or hydrolyzable tannins which can play important roles in plant structural integrity and defense against biotic and abiotic stresses (Vogt, 2010). On the other hand, specific Cd induction of the “Glutathione metabolism” and “Sesquiterpenoid and triterpenoid biosynthesis” pathways in mycorrhizal roots suggest an increase in the biosynthesis of antioxidant compounds. Activation of the antioxidant GSH metabolism in mycorrhizal roots exposed to Cd may explain the very low proportion of up-regulated stress-related proteins, when compared to non-mycorrhizal *V. myrtillus* roots. The much higher proportion of HSPs in non-mycorrhizal *V. myrtillus* roots may reflect the higher Cd content. A similar up-regulation of the GSH biosynthetic pathway has been reported in AM plants exposed to Cd (Huang et al., 2017; Shi et al., 2019), and it was suggested by these authors that AM fungi might attenuate Cd stress by enhancing the production of thiolic compounds in AM roots.

In plants, phytochelatin synthase uses GSH as substrate to generate phytochelatins that, together with metallothioneins, can bind heavy metals and thus reduce metal toxicity in the plant cell (Cobbett and Goldsbrough, 2002; Clemens, 2006b). Phytochelatin- and GSH-metal complexes can be then sequestered in the vacuole by specific ABC transporters on the tonoplast (Cobbett and Goldsbrough, 2002). Transcripts coding for both phytochelatin synthases and metallothioneins could be identified in *V. myrtillus*, but their expression was not significantly different in any of the pairwise comparisons (data not shown). A similar result was reported by Ouziad et al. (2005), who investigated heavy metal accumulation and differential gene expression in AM tomato grown under heavy metal stress. However, the activity of phytochelatin synthase is post-transcriptionally regulated (Cobbett, 2000; Gautam et al., 2012), and we therefore cannot exclude that phytochelatins might contribute to alleviate Cd toxicity.

We observed, only in mycorrhizal *V. myrtillus* exposed to Cd, a significant and specific down-regulation of important copper (Cu) binding proteins. Among them were Cu/Zn superoxide dismutase, a ubiquitous and highly conserved antioxidant enzyme (Apel and Hirt, 2004) and its copper chaperone. Other Cu binding proteins significantly down-regulated in Cd exposed mycorrhizal *V. myrtillus* were laccases, cupredoxins, and glyoxal oxidases. Cupredoxins belong to the phytocyanin family and likely function as electron−transfer shuttles between proteins (Choi and Davidson, 2011), whereas laccases are multi-copper oxidases involved in oxidative polymerization of lignin (Schuetz et al., 2014) and other substrates (Turlapati et al., 2011). Glyoxal oxidases generate H_2_O_2_, which is used by plant peroxidases for the polymerization of lignin and the cross-linkage of apoplastic structural protein. Interestingly, most transcripts coding for these Cu binding proteins are known targets of specific plant microRNAs. MicroRNAs (miRNAs) are important regulators in plant development and plant response to stress (Voinnet, 2009; Khraiwesh et al., 2012; Rogers and Chen, 2013), and a specific role has been demonstrated in response to metal toxicity (Mendoza-Soto et al., 2012; Gupta et al., 2014). In particular, laccases and cupredoxins are targets of *miR408* (Fang et al., 2013; Ma et al., 2015) and Cu/Zn superoxide dismutase, as well as its copper chaperone, are targeted by *miR398* (Sunkar et al., 2006; Beauclair et al., 2010). *MiR408* and *miR398* are regulated by the same transcription factor SPL7 and are members of the copper miRNAs, a group of small RNAs involved in Cu homeostasis (Pilon, 2017). Through their regulatory function on Cu-binding proteins, these miRNAs may modulate Cu availability in the cell by reallocating intracellular Cu. Copper is an essential metal involved in many processes in plants, and Cu uptake and reallocation have been demonstrated to play a key role in cadmium resistance in *Arabidopsis* (Gayomba et al., 2013; Carrio-Segui et al., 2015). We currently don’t know whether the down-regulation of some Cu-binding proteins in mycorrhizal *V. myrtillus* exposed to Cd is related to their biological function or to Cu reallocation, but it is intriguing to note that a copper transporter (COPT) similar to the *A. thaliana* COPT3 was specifically induced in Cd-exposed mycorrhizal roots. In *Arabidopsis*, COPT3 is located in internal membranes and may function in Cu mobilization from intracellular stores (Burkhead et al., 2009). Given the pivotal role of miRNAs in regulating the plant response to Cd, it would be important to understand if the mycorrhizal association may change the expression of specific miRNAs in *V. myrtillus*.

### *O. maius* Responses to Cd Exposure

No differences in fungal biomass were found when *O. maius* Zn was grown as free-living mycelium on control or Cd amended medium, which is not surprising considering the metal tolerant phenotype described for this ERM fungus (Perotto et al., 2012; Daghino et al., 2016) and the relatively low Cd concentration (1 μM) used. Reduced *O. maius* Zn growth was in fact recorded at 0.05 mM Cd (Chiapello et al., 2015), a concentration 50 fold higher than the one used in the present study.

Although Cd had no significant effects on fungal growth *in vitro*, 120 up-regulated genes (FC > 2) were identified in the free-living mycelium exposed to Cd, as compared with the control medium. Among these genes were hydrolases and oxidoreductases, enzyme categories known to be involved in metal tolerance (Patra et al., 1994; Lebrun et al., 2010). In particular, fungal hydrolases may play a positive role in metal tolerance because they would increase the ability of fungi to obtain nutrients in adverse environments (Moreno et al., 2012).

We also unexpectedly found a high percentage (11.76%) of specifically up-regulated Small Secreted Proteins (SSPs) coding genes. SSPs are a heterogeneous class of proteins that have in common the small size (<300 amino acids) and a signal peptide. SSPs have been mostly studied as effector proteins in pathogenic and mutualistic plant–microbe interactions (Martin, 2010; Plett et al., 2011). Up-regulation of some SSPs by Cd represents an interesting observation and opens up new perspectives in the study of SSPs functions unrelated to biotic interactions. Indeed, fungi with different lifestyles and ecological strategies feature SSPs, suggesting that SSPs functions can lie outside plant–fungal interactions (Feldman et al., 2017; Valette et al., 2017). A potential involvement of SSPs in increasing fungal tolerance to toxic compounds released during substrate degradation (such as aromatic compounds or reactive oxygen species) has been suggested for some saprotrophic fungi (Valette et al., 2017). In mycorrhizal fungi, Doré et al. (2015) reported for the first time that some SSPs produced by the ECM fungus *Hebeloma cylindrosporum* may be involved in environmental stress response rather than symbiosis. Thus, it would be interesting to further investigate, in ERM fungi, the role of SSPs in heavy metal tolerance.

Fungal transporters belonging to the Major Facilitator Superfamily (MFS) represented 52% of the total TP genes regulated by Cd, more than 50% of them corresponding to fungal transcripts up-regulated in symbiotic conditions. The MFS, one of the largest families of membrane transporters, are permeases that move a wide variety of organic and inorganic molecules across biological membranes, such as amino acids, sugars, nitrate and phosphate (Yan, 2015). A role for the MFS in fungal metal tolerance was also reported by Majorel et al. (2014) who suggest a role for a MFS permease in nickel resistance in the ECM fungus *Pisolithus albus*, and by Xu et al. (2015), who reported for *Penicillium janthinellum* a crucial role of a MFS in the solute compartmentalization transport related to Cu resistance.

### Cd Stress Specifically Influences Gene Expression of Putative Transporters in *V. myrtillus*

Membrane transporters (TPs) are the main responsible of metal/ion and nutrient translocation/homeostasis across cell membranes and likely play an important role in Cd accumulation in the cell. Interestingly, Cd induced a higher number of putative TPs in mycorrhizal than in non-mycorrhizal *V. myrtillus* roots. Among the TPs up-regulated by Cd in non-mycorrhizal *V. myrtillus* roots, most were identified as ABC transporters. The ABC superfamily contains both uptake and efflux transport systems, having dozens of sub-families linked to substrate specificity (Pang et al., 2013). In particular, most transcripts up-regulated by Cd in non-mycorrhizal *V. myrtillus* roots were members of the ABC family with homology to the *Arabidopsis* Pleiotropic Drug Resistance (PDR) transporter. This ABC sub-family is related to metal tolerance and detoxification in plants. For example, the AtPDR8/AtABCG32 and AtPDR12/AtABCG40 in *Arabidopsis* are induced by Cd and Pb and they could confer resistance to the plant by pumping these cations out of the plasma membrane in case of metal surplus (Nuruzzaman et al., 2014). However, most ABC transporters were not differentially regulated in mycorrhizal and non-mycorrhizal *V. myrtillus* roots exposed to Cd and, unless a post-translational control occurs, it is unlikely that they are responsible for the lower Cd content observed in mycorrhizal roots.

Six oligopeptide transporters (OPT) were strongly up-regulated in mycorrhizal roots exposed to Cd, the two most up-regulated being similar to OPT 9 of *A. thaliana*, involved in peptide translocation across the cellular membrane, and the other four belonging to the Metal-nicotianamine transporter *Yellow Stripe like 3* (YSL3) type. The OPT family includes transporters for at least three types of substrates: small peptides, amino acids bound to metals and glutathione (see Lubkowitz, 2011). OPTs involved in GSH transport may play direct as well as indirect roles in many GSH-mediated processes, including Cd tolerance. For example, expression of BjGT1 is altered by environmental Cd levels (Srivastava et al., 2010). Despite their interesting expression profile, we currently do not know the substrates transported by the two most up-regulated OPTs in *V. myrtillus* mycorrhizal roots. Given the increased GSH metabolism observed in mycorrhizal roots exposed to Cd, it is possible that these up-regulated OPTs are involved in GSH-mediated processes.

Many regulated transcripts in mycorrhizal and non-mycorrhizal plants exposed to Cd belong to the NRAMP transporter family. NRAMPs are highly conserved proteins that function as proton/metal symporters, with a broad spectrum of metal cation substrates (Nevo and Nelson, 2006). In plants, these TPs families facilitate the acquisition of iron and manganese from soil, but they can also transport Cd (Cailliatte et al., 2010; Sasaki et al., 2012). NRAMPs on internal cell membranes are likely involved in intracellular metal sequestration and distribution (Cailliatte et al., 2009), while NRAMPs localized on the plasma membrane were found to be responsible for Cd uptake and accumulation in rice (Takahashi et al., 2011; Sasaki et al., 2012). Cd exposure up-regulated a Nramp3-like gene in both mycorrhizal and non-mycorrhizal *V. myrtillus* roots, whereas only in mycorrhizal *V. myrtillus* roots it strongly down-regulated three members of the NRAMP family similar to *A. thaliana* Nramp1 and Nramp6. In *A. thaliana*, NRAMP1 functions in Cd uptake (Cailliatte et al., 2009), and we could speculate that its transcriptional down-regulation in *V. myrtillus* may lead to a decreased influx of Cd in mycorrhizal roots, resulting in the lower Cd content observed. However, the cellular localization of these regulated NRAMP transporters and their exact role in *V. myrtillus* remains to be established.

Another plant TP that may be involved in reducing Cd content in the mycorrhizal *V. myrtillus* root tissues was identified as a member of the Cadmium tolerance Efflux Pump (CTEP) Family. Proteins of this family are exclusively found in plants, and the gene corresponding to this TP (transcript ID TRINITY_DN46689_c2_g1_i2) was specifically up-regulated in mycorrhizal roots exposed to Cd. Experiments in wheat seedlings demonstrated that expression of TaTM20, a CTEP gene from *Triticum aestivum*, was induced by Cd treatments and reduced subsequent root Cd accumulation (Kim et al., 2008).

## Conclusion

Cadmium is one of the most serious metal pollutants and strongly inhibits plant growth and development. The mycorrhizal symbiosis can increase plant Cd tolerance, although the mechanisms are far from being understood. Here, we showed that ericoid mycorrhizal roots of *V. myrtillus* exposed to Cd have a reduced metal content when compared with non-mycorrhizal plants. Transcriptomic data outlined the regulation in symbiosis of some plant genes that indicate alleviation of Cd stress, such as those involved in GSH metabolism. Some plant metal transporters known to transport Cd were also regulated in symbiosis and may be responsible for the reduced Cd content observed in mycorrhizal roots exposed to this metal. Further investigations are of course needed to better understand the localization and the potential roles of these plant transporters.

An intriguing observation was the concerted and significant reduction, in mycorrhizal roots exposed to Cd, of transcripts known to be targets of plant Cd-responsive microRNAs (e.g., Ding et al., 2011; Fang et al., 2013; Xu et al., 2013). In addition to copper binding proteins, some transporters of the Nramp family are also gene targets of specific miRNAs, namely the Cd-responsive *miR167* (Zhou et al., 2012; Meng et al., 2017). MiRNA play an important regulatory role in plant response to heavy metals, and knowledge of the influence of mycorrhizal fungi on miRNA expression may provide novel insight into the protective role of mycorrhizal fungi toward their host plant.

## Data Availability Statement

The datasets generated for this study are accessible through the NCBI’s GEO Series accession numbers GSE119266 and GSE119554.

## Author Contributions

SC, EMa, EMo, AK, FM, and SP planned and designed the research. SC, EMa, EMo, AK, SD, and SP performed experiments, collected, analyzed, or interpreted data. H-RK prepared RNA samples for the RNA-Seq experiments. CM performed single and double hierarchical clustering analyses. KB and EL performed RNA-Seq experiments. SP, EMa, and SC wrote the manuscript.

## Conflict of Interest

The authors declare that the research was conducted in the absence of any commercial or financial relationships that could be construed as a potential conflict of interest.

## Footnotes

1 https://genome.jgi.doe.gov/mycocosm/home

2 https://www.arabidopsis.org/

3 http://www.tcdb.org
