# Supplementary material for: Modulation of Plant and Fungal Gene Expression Upon Cd Exposure and Symbiosis in Ericoid Mycorrhizal Vaccinium myrtillus
Source: Front Microbiol. 2020 Mar 9;11:341. doi: 10.3389/fmicb.2020.00341 (PMC7075258; doi:10.3389/fmicb.2020.00341)
Supplement: Supplementary file 1 [file Data_Sheet_1.docx]

Supplementary Material

Modulation of plant and fungal gene expression upon Cd exposure and symbiosis in ericoid mycorrhizal *Vaccinium myrtillus*

Salvatore Casarrubia^§^, Elena Martino^§^, Stefania Daghino, Annegret Kohler, Emmanuelle Morin, Hassine-Radhouane Khouja, Claude Murat, Kerrie W. Barry, Erika A. Lindquist, Francis M. Martin, Silvia Perotto^*^

*** Correspondence:** Silvia Perotto: [silvia.perotto@unito.it](mailto:silvia.perotto@unito.it)

Elena Martino: [elena.martino@unito.it](mailto:elena.martino@unito.it)

§ These authors equally contributed to the work

# Supplementary Figures and Tables

**
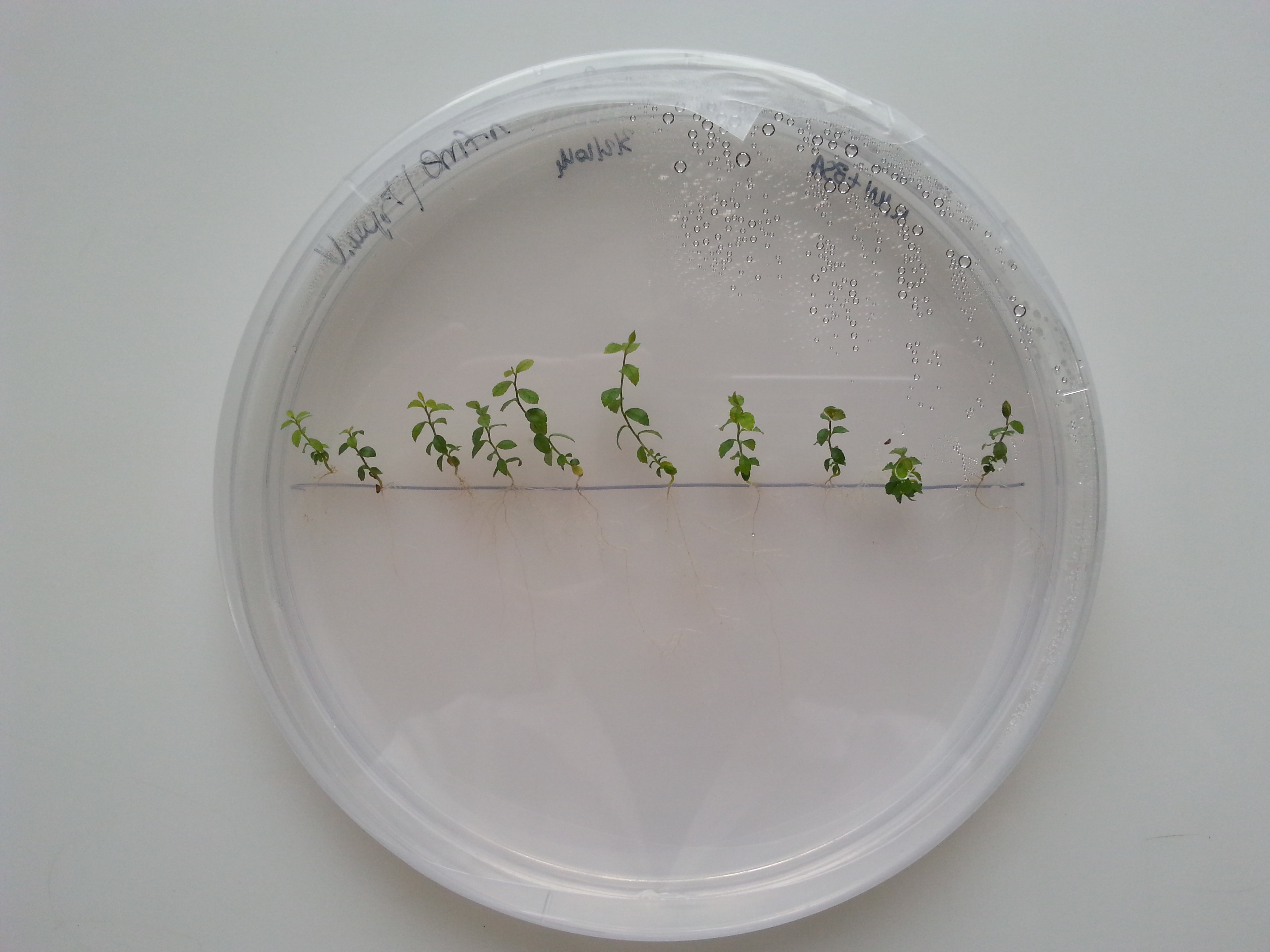
**

**Supplementary Figure S1.** ***V. myrtillus* seedling *in vitro* cultivation system.** *V. myrtillus* seedlings were grown *in vitro* for 1.5 months in the presence and in the absence of 1 µM Cd and *O. maius* Zn inoculum.

**
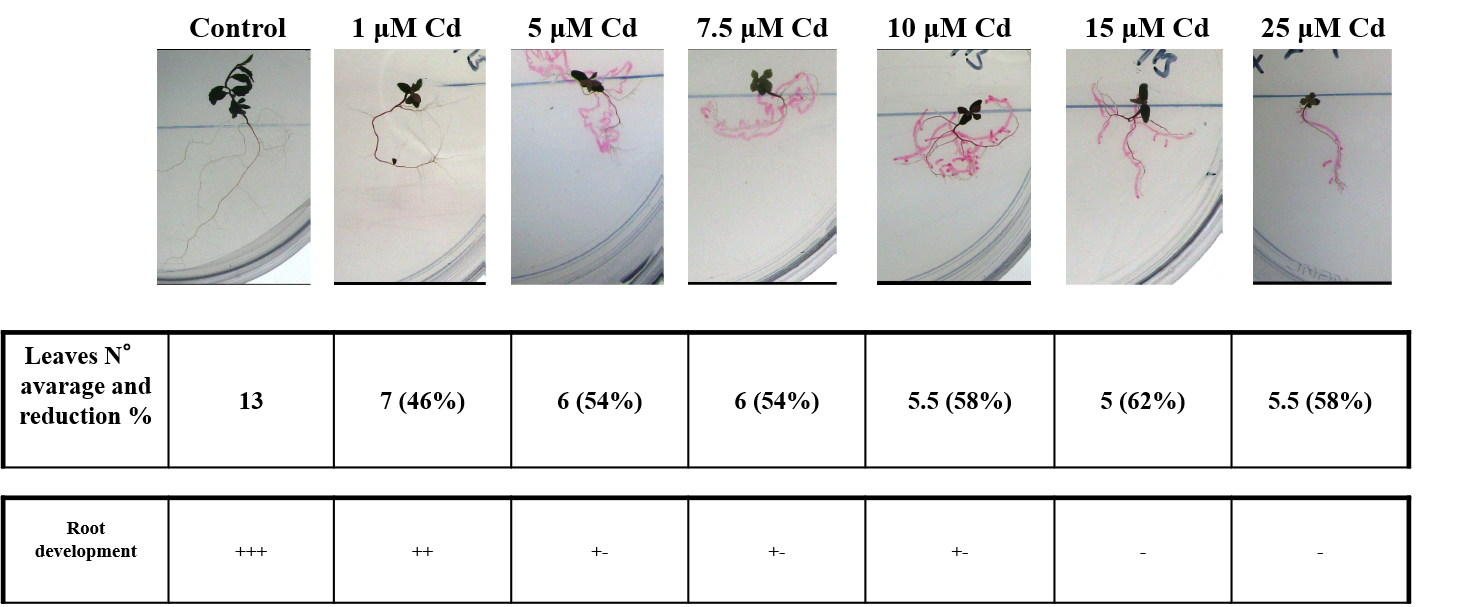
**

**Supplementary Figure S2. Growth tests on different Cd concentrations of *V. myrtillus* plants.** *V. myrtillus* plants were grown on control culture medium and on culture medium added with 1, 5, 7.5, 10, 15 and 25 µm Cd. The average number of leaves counted per plant for each condition as well as the percentage of leaves number reduction and the evaluation of root development are also reported.

**
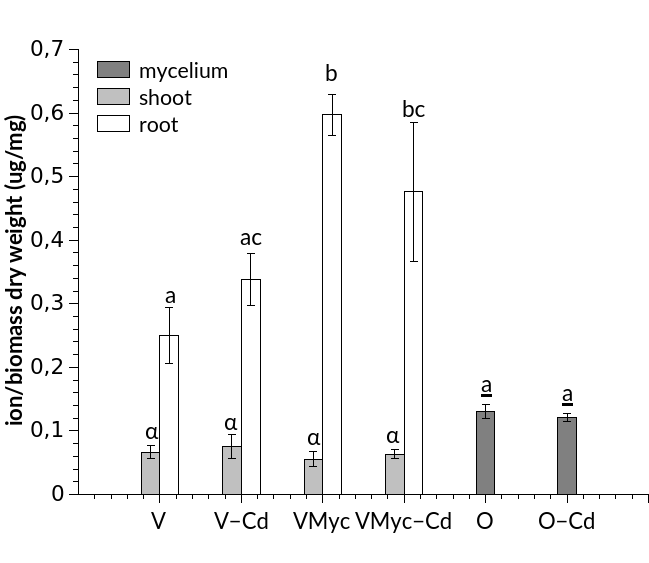
**

**Supplementary Figure S3. Fe concentrations in *V. myrtillus* roots and shoots and in *O. maius* Zn*.*** Fe concentrations were quantified in the roots and in the shoots of mycorrhizal and non-mycorrhizal *V.* *myrtillus* seedlings and in *O. maius* Zn mycelium, grown under control and Cd-stressed conditions. Bars represent the mean ±SD, n=4. Different letters (greek letters for shoots, latin letters for roots, latin underlined letters for mycelium) indicate statistically significant differences (*p < 0.05*) according to ANOVA with Tukey’s as post hoc test.

**
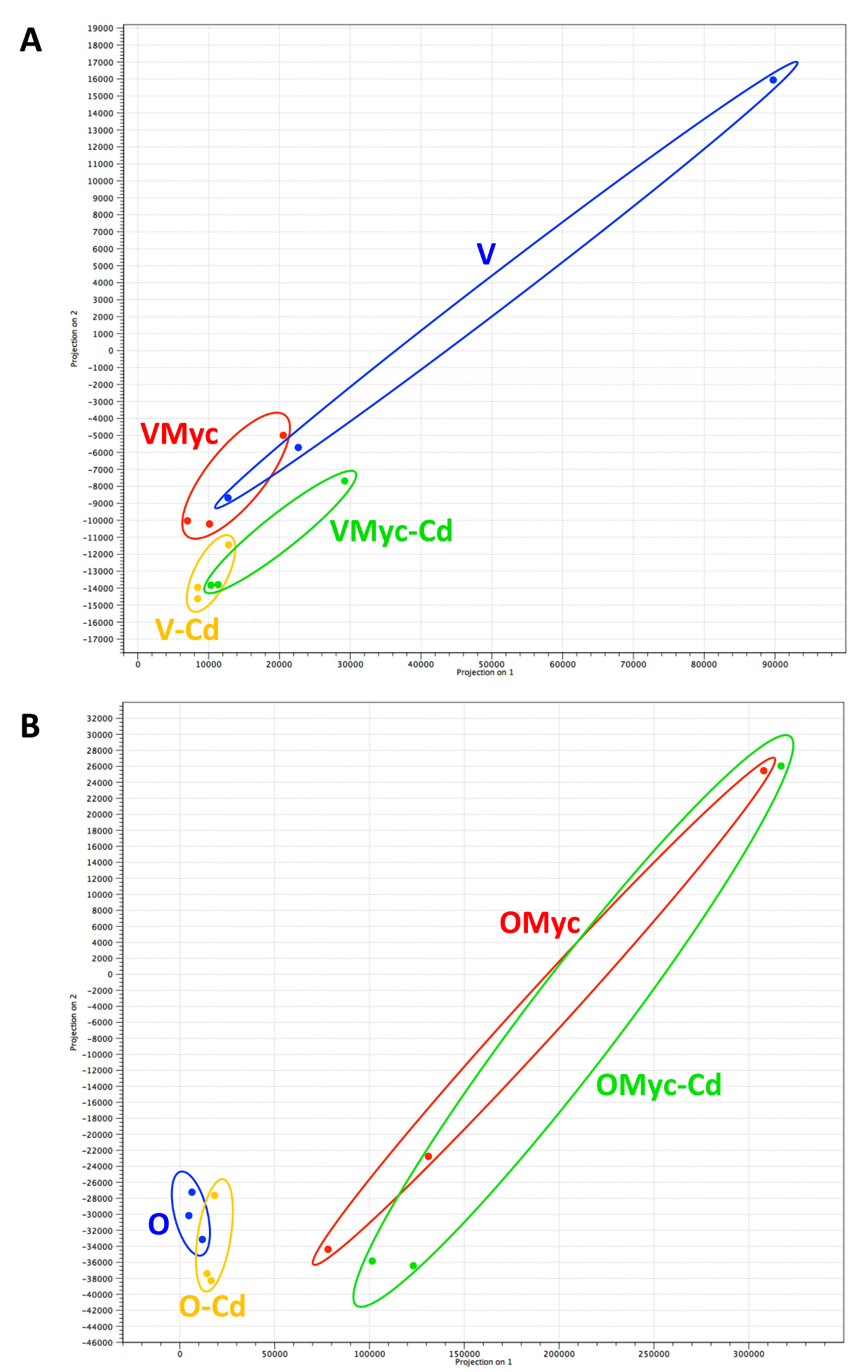
**

**Supplementary Figure S4. Principal Component Analysis (PCA) of *O. maius* Zn and *V. myrtillus* transcriptomes.** PCA analysis of *V. myrtillus* (A) and *O. maius* Zn (B) transcriptome data sets. Biological replicates of the different experimental conditions were indicated with different colors. A: *V. myrtillus* roots exposed (V-Cd) or not (V) to 1 µM Cd; *V. myrtillus* mycorrhizal roots exposed (VMyc-Cd) or not (VMyc) to 1 µM Cd. B: *O. maius* Zn free living mycelium exposed (O-Cd) or not (O) to 1 µM Cd; *O. maius* Zn colonizing *V. myrtillus* roots exposed (OMyc-Cd) or not (OMyc) to 1 µM Cd.

**Supplementary Figure S5. KEGG enrichment analysis of *V. myrtillus* and *O. maius* Zn regulated genes (p<0.05).** A list of significantly enriched biochemical and metabolic pathways found in the KEGG Pathway database was generated for *V. myrtillus* (V) and for *O. maius* Zn (O) regulated genes (*p<0.05*) considering the three pairwise comparisons (see Table S1 for acronym explanation). The darker part of the histograms represent the number of transcripts belonging to each specific KEGG class over the number of all annotated and regulated transcript/genes in the given comparison. The values reported near each histogram are p. adjust values, which were corrected according to the Beniamini-Hochberg test.

**
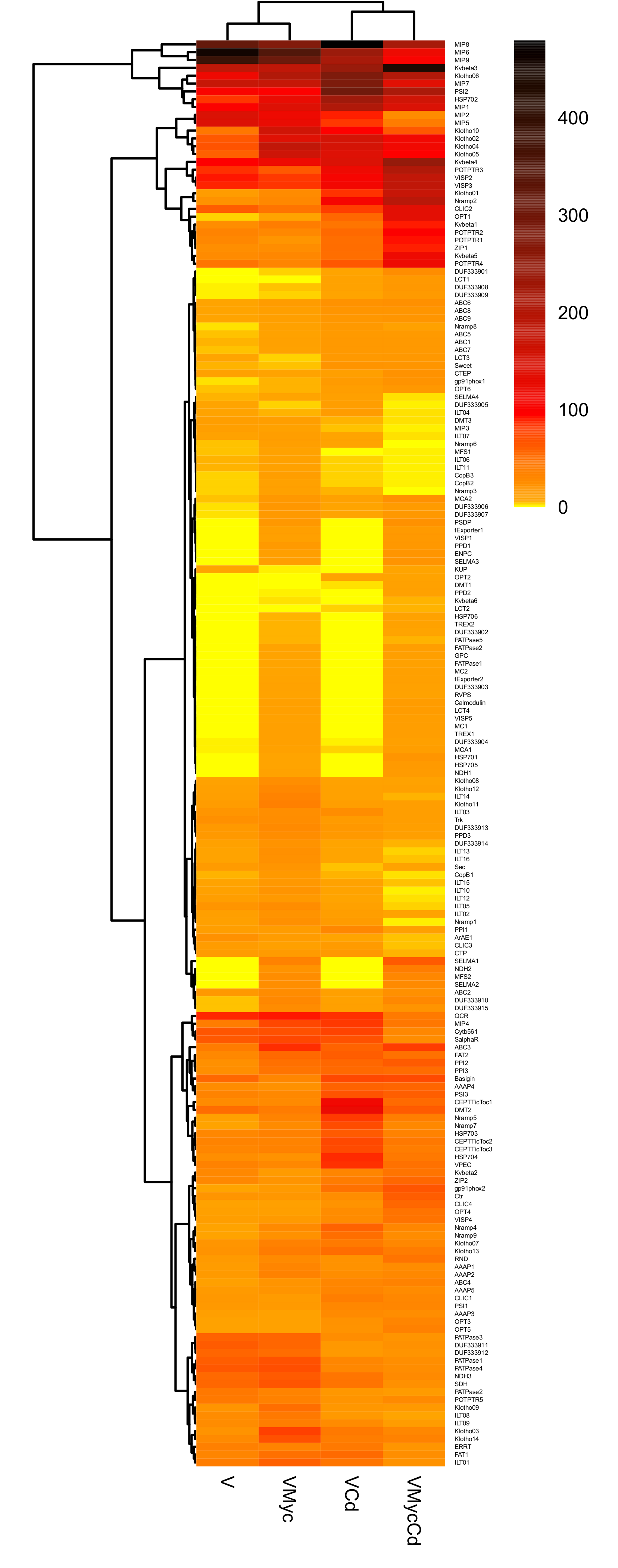
**

**Supplementary Figure S6. Cd-exposure and mycorrhizal effects on the expression pattern of *V. myrtillus* putative TPs families.** Double hierarchical clustering of the regulated (*p<0.05*) putative TPs of *V. myrtillus*. Rows are grouped according to the expression profile, whereas columns are ordered by the condition hierarchical clustering. The color is representative of the mean expression value of each transcript (see tif image 1).

**
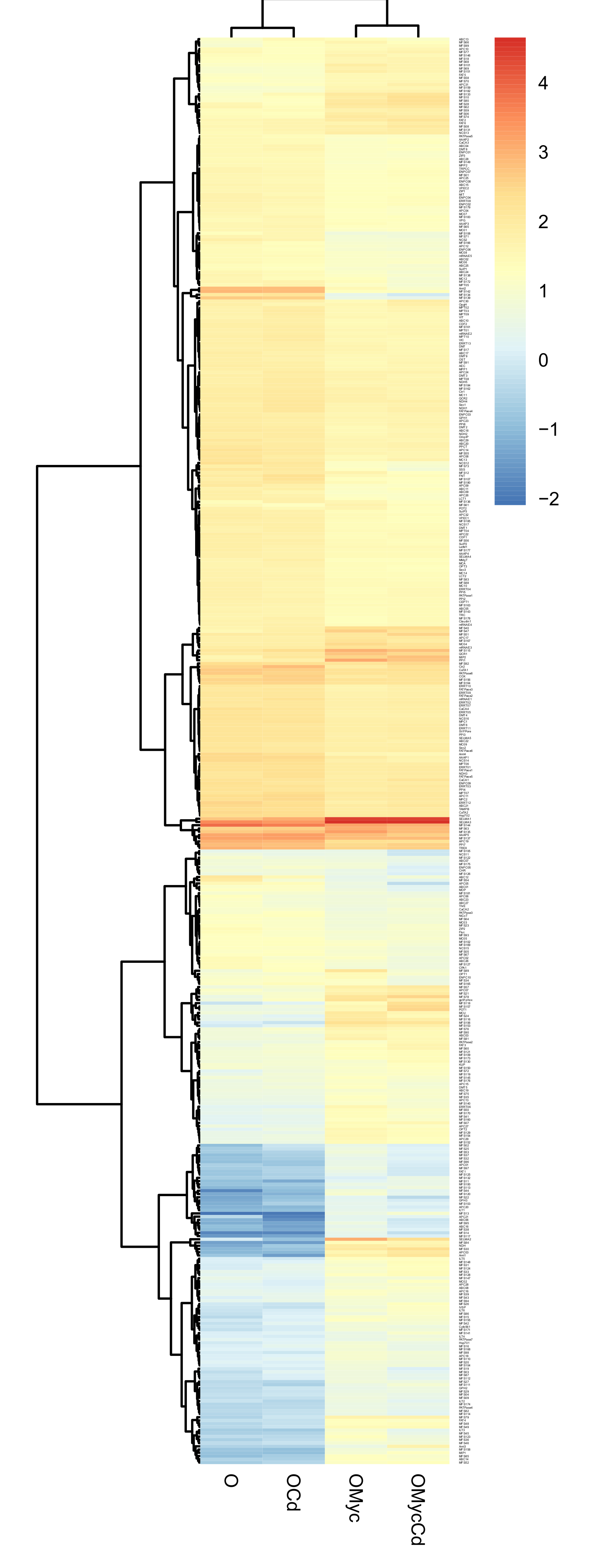
**

**Supplementary Figure S7. Cd-exposure and mycorrhizal effects on the expression pattern of *O. maius* Zn putative TPs families.** Double hierarchical clustering of the regulated (*p<0.05*) putative TPs of *O. maius* Zn. Rows are grouped according to the expression profile, whereas columns are ordered by the condition hierarchical clustering. The color is representative of the Log_10_ of mean expression value of each transcript (see tif image 2).

## Supplementary Tables

**Table S1. E**xperimental comparisons considered for *O. maius* and *V. myrtillus* in the analysis of the RNAseq data, with acronym explanation.

| **Organism** | **Comparison** | **Brief description** |
| --- | --- | --- |
| *V. myrtillus*  (V) | V-Cd vs V | Plant genes regulated by Cd in the free living status |
|  | VMyc vs V | Plant genes regulated by symbiosis on control medium |
|  | VMyc-Cd vs VMyc | Plant genes regulated by Cd in the mycorrhizal status |
| *O. maius* Zn (O) | O-Cd vs O | Fungal genes regulated by Cd in the free living status |
|  | OMyc vs O | Fungal genes regulated by symbiosis on control medium |
|  | OMyc-Cd vs OMyc | Fungal genes regulated by Cd in the mycorrhizal status |

**Table S2. Percentage of regulated transcripts in the *V. myrtillus* *de novo* assembly.** Homology of *V. myrtillus* transcripts with the *A. thaliana* and the *V. macrocarpon* annotated genomes after *de novo* assembly. The number and percentage of transcripts with significant p-values (*p<0.05*) in the different comparisons that found a match with the two reference genomes are indicated (see Table S1 for acronym explanation).

|  | **Total n° of**  ***V. myrtillus* contigs p<0.05** | **Total n° of matches on**  ***A. thaliana* annotated transcripts** | **%**  ***A. thaliana* hits** | **Total n° of matches on**  ***V. macrocarpon* annotated transcripts** | **%**  ***V. macrocarpon* hits** |
| --- | --- | --- | --- | --- | --- |
| V-Cd vs V | 439 | 227 | 51.71% | 216 | 49.20% |
| VMyc vs V | 471 | 217 | 46.07% | 207 | 43.95% |
| VMyc-Cd vs VMyc | 876 | 453 | 51.71% | 459 | 52.40% |

**Table S3.** List of *V. myrtillus* and *O. maius* Zn significantly regulated genes (see Excel file)

**Table S4.** List of *V. myrtillus* putative transporters (see Excel file)

**Table S5.** List of *O. maius* Zn putative transporters (see Excel file)
